# Indomethacin promotes browning and brown adipogenesis in both murine and human fat cells

**DOI:** 10.1002/prp2.592

**Published:** 2020-05-19

**Authors:** Haley Overby, Yang Yang, Xinyun Xu, Shu Wang, Ling Zhao

**Affiliations:** ^1^ Department of Nutrition The University of Tennessee Knoxville TN USA; ^2^ Department of Nutritional Sciences Texas Tech University Lubbock TX USA

**Keywords:** brown adipogenesis, browning, indomethacin, PPARγ

## Abstract

Indomethacin (Indo), a nonsteroidal antiinflammatory drug, has been shown to promote murine brown adipogenesis both in vitro and in vivo, possibly due to its peroxisome proliferator‐activated receptor gamma (PPARγ)‐agonist activities. However, it is unclear whether Indo induces browning of white adipocytes from both murine and human origins or induces human brown adipogenesis. To bridge the gap, this study investigated the effects of increasing concentrations of Indo on murine 3T3‐L1, human primary subcutaneous white adipocytes (HPsubQ), and human brown (HBr) adipocytes. The results show that Indo dose‐dependently enhanced 3T3‐L1 adipocyte differentiation and upregulated both mRNA and protein expression of brown and beige adipocyte markers, while simultaneously suppressing white adipocyte‐specific marker mRNA expression. mRNA and protein expression of mitochondrial biogenesis and structural genes were dose‐dependently enhanced in Indo treated 3T3‐L1 adipocytes. This was accompanied by augmented mitochondrial DNA, enhanced oxygen consumption, proton leak, and maximal and spare respiratory capacity. Dose‐dependent transactivation of PPARγ confirmed Indo's PPARγ‐agonist activity in 3T3‐L1 cells. Knockdown of PPARγ significantly attenuated Indo's activities in selective browning genes, demonstrating PPARγ dependence of these effects. Moreover, Indo enhanced mRNA and protein expression of brown markers in HPsubQ adipocytes. Interestingly, Indo‐induced differential effects on individual PPARγ isoforms with significant dose‐dependent induction of PPARγ‐2 and suppression of PPARγ‐1 protein expression. Finally, Indo significantly promoted brown adipogenesis in HBr cells. Taken together, these results demonstrate Indo to be a potent thermogenic compound in both murine and human fat cells and may be explored as a therapeutic agent for obesity treatment and prevention.

**Abbreviations**
ADRB3: adrenergic receptor β3
BAT: brown adipose tissue
C/EBP: CCAAT enhancer binding protein alpha
CIDEA: cell death inducing DFFA like effector a
COX: cyclooxygenase
CYTC: Cytochrome c
IGFBP3: insulin growth factor binding protein 3
ISO: isoproterenol
MNSOD: manganese containing superoxide dismutase
OCR: oxygen consumption rate
ORO: oil red O
PGC1α: peroxisome proliferator‐activated receptor gamma coactivator 1‐alpha
PLIN: perilipin
PPAR: peroxisome proliferator‐activated receptor
PRDM16: PR Domain‐containing 16
Rosi: rosiglitazone
TZD: thiazolidinedione
UCP1: uncoupling protein 1
VDAC1: voltage‐dependent anion‐selective channel 1
WAT: white adipose tissue

## 1 INTRODUCTION

Energy‐dense diets and sedentary lifestyles are primary contributing factors to the still‐growing obesity epidemic facing the United States and worldwide.^1^, ^2^ Obesity‐related conditions such as diabetes, heart disease, and cancer are among the leading causes of preventable deaths in America.^1^ Advocating for improved diet and lifestyle factors while also increasing physical activity is always the first line of treatment. However, these pursuits are difficult to maintain and may not be feasible for ill or disabled individuals. Thus, it is imperative to develop novel strategies for obesity treatment and prevention that will be effective for everyone.

Promoting brown adipogenesis of brown adipose tissue (BAT) and browning of white adipose tissue (WAT) are considered novel approaches to combat obesity and associated metabolic disorders.^3^, ^4^ BAT is a highly vascularized, mitochondria‐enriched tissue that is capable of burning fat and producing heat via uncoupling protein 1 (UCP1)‐mediated dissipation of the proton gradient.^5^, ^6^ Peroxisome proliferator‐activated receptor gamma (PPARγ) is upregulated in brown fat and its activation triggers a series of downstream signaling events, leading to increased UCP1 and PPARγ‐coactivator 1‐alpha (PGC1α) expression, resulting in increased thermogenesis and mitochondrial biogenesis which ultimately leads to increased energy expenditure.^7^ Pharmacological or nutritional strategies targeted to enhance BAT activity, mass, and function can be an effective attempt to restore energy balance, promote fat loss, and improve the metabolic profile in obesity.^8^, ^9^, ^10^

Similar to brown adipocytes, beige or brite (“brown in white”) adipocytes generated by browning are characterized by their multilocular lipid droplet morphology, larger volume of functional mitochondria, and expression of brown fat marker genes, such as UCP1, PPARγ, PGC1α, PR Domain‐containing 16 (PRDM16), and cell death‐inducing DFFA‐like effector a (CIDEA).^8^, ^9^, ^10^ These UCP1 expressing adipocytes are formed within WAT in response to cold exposure, β‐adrenergic receptor (β‐AR) agonists (eg, norepinephrine, isoproterenol), and some nutritional and pharmacological agents.^3^, ^8^, ^11^

Indomethacin (Indo) is a nonselective cyclooxygenase (COX)‐1/COX‐2 inhibitor and is used as an FDA approved drug to treat and relieve acute pain and inflammatory conditions.^12^ Several NSAIDs, including Indo, have been shown to have off‐target effects, such as activation of PPARγ.^13^, ^14^, ^15^ However, Indo is distinct from other NSAIDs in that it acts as a strong *partial* PPARγ agonist with activities similar to that of the well‐known PPARγ‐agonist, rosiglitazone (Rosi).^13^, ^15^ Rosi and other thiazolidinedione (TZD) analogs have been used as insulin‐sensitizing drugs in the treatment of type 2 diabetes mellitus (T2DM).^16^ Rosi is commonly used in cell culture to promote adipogenesis as well as induce browning of white adipocytes.^17^, ^18^, ^19^, ^20^ It is postulated that Rosi's browning effect is a result of its PPARγ‐agonist activity.^18^, ^21^ Therefore, having ability to act as a strong *partial* PPARγ agonist makes Indo of particular interest in the hunt for novel browning agents.

It has been previously demonstrated that Indo promotes brown adipogenesis in murine brown adipocytes in vitro as well as in vivo in the BAT of C57Bl/6J mice when Indo was delivered via osmotic pumps.^22^ As of yet, it has not been demonstrated whether Indo could induce browning of white adipocytes and whether it could have similar effects on human fat cells. Therefore, to bridge the gap, the current study investigated the effects of Indo on differentiating murine and human white fat cells and human brown fat cells.

## 2 MATERIALS AND METHODS

### 2.1 Reagents

Dexamethasone (Dex), 3‐Isobutyl‐L‐methylxanthine (IBMX), insulin, triiodothyronine (T_3_), Rosi, and isoproterenol (ISO) were purchased from Sigma‐Aldrich. Indo was purchased from Cayman Chemical. Dulbecco's modified eagle's medium (DMEM) and calf serum (CS) were purchased from Hyclone and fetal bovine serum (FBS) from Atlanta Biologicals. Penicillin and streptomycin (P/S) were purchased from ThermoFisher Scientific. Antibodies for UCP1 (Product # U6382), β‐AR3 (Product # SAB4500584), and β‐Actin (Product # A1978) were purchased from Sigma‐Aldrich and PGC1α antibody (Catalog # AB3242) was from Millipore Sigma. Antibodies for voltage‐dependent anion‐selective channel 1 (VDAC1) (Catalog # 98708), Cytochrome c (CYTC) (Catalog # 13156), and the manganese containing superoxide dismutase (MNSOD) (Catalog # 137254) were from Santa Cruz Biotechnology, and Antibodies for ERK1/2 (Catalog # 4695), PPARγ (Catalog # 2443), Perilipin (Catalog # 9349), and anti‐rabbit IgG horse‐radish peroxidase (HRP)‐conjugated antibody (Catalog # 7074) were from Cell Signaling Technology.

### 2.2 Cell culture, induction of adipocyte differentiation, and treatments

#### 2.2.1 3T3‐L1 preadipocyte maintenance and differentiation

3T3‐L1 differentiation was performed as previously described.^23^ Murine 3T3‐L1 fibroblasts (ATCC) were grown in DMEM containing 10% CS in a 5% CO_2_, 37°C environment until confluence. The cells were then induced to differentiate with DMEM containing 10% FBS, 1 μmol/L Dex, 0.5 mmol/L IBMX, and 10 μg/mL insulin for 3 days. The differentiating cells were then maintained with DMEM containing 10% FBS and 10 μg/mL insulin for an additional two days until day 5 when the media was changed to DMEM containing only 10% FBS until day 7. On day 7 the cells were stimulated with β‐AR‐agonist, ISO (1 μmol/L), or the vehicle control (DMSO) for 6 hours (for mRNA and mitochondrial DNA (mtDNA) analysis) or 24 hours (for western blot) to activate beige adipocytes. Indo, Rosi, and vehicle controls were added fresh during each change of the media, Indo was used at concentrations of 0, 2, 5, 10, 20, and 50 μmol/L. PPARγ‐agonist, Rosi (1 μmol/L), was used as the positive control.

#### 2.2.2 Human primary subcutaneous adipocyte differentiation

Human primary preadipocytes from subcutaneous adipose tissue (HPsubQ) originating from six female obese, nondiabetic donors (Lot: SL0031) and the media used to grow, differentiate, and maintain these cells were obtained from ZenBio. Cells were maintained and differentiated for 14 days, according to the manufacturer's instructions. Indo, Rosi, and vehicle controls were added fresh during each change of the media, Indo was used at concentrations of 0, 5, 10, and 20 μmol/L. Rosi (1 μmol/L) was used as the positive control.

#### 2.2.3 Human brown preadipocyte line differentiation

The human brown preadipocyte line (HBr) was a gift from Dr Yu‐hua Tseng at Joslin Diabetes Center.^24^ Cells were grown and differentiated according to the provider's instructions. Briefly, HBr preadipocytes were grown in DMEM containing 10% FBS in a 5% CO_2_, 37°C environment until confluence. The cells were then induced to differentiate with DMEM containing 10% FBS, 0.5 mmol/L IBMX, 33 μmol/L biotin, 17 μmol/L pantothenate, 0.5 μmol/L insulin, 0.1 μmol/L Dex, and 2 nmol/L T3 for 3 weeks. Treatments were added fresh to each change of media, which was changed every 2‐3 days for a total of 21 days prior to lysis for further analysis.

### 2.3 Cell viability

Cell viability was measured via the colorimetric MTT (3‐(4,5‐dimethylthiazolyl‐2)‐2,5‐diphenyltetrazolium bromide) metabolic activity assays (Research Products International). 3T3‐L1 cells were seeded in 48‐well plates to reach 50%‐70% confluence the following day. The cells were then treated with varying concentrations of Indo (0, 2, 5, 10, 20, and 50 μmol/L) for 24 hours. Then MTT reagent was added to cell media in a 1:5 ratio in dark conditions for a 3 hours incubation at 37°C, after which time the media was removed, and the insoluble formazan was eluted in 60% isopropanol. After incubating the cells with isopropanol for 5 minutes, a portion of the formazan‐solution was added to a clear 96‐well plate where absorbance was measured at 570 nm using GloMax‐Multi Detection System (Promega). These results were normalized with cell lysate total protein concentration using bicinchoninic acid (BCA) Protein Assay Kit (ThermoFisher Scientific).

### 2.4 ORO staining and quantification

Lipid accumulation in differentiated 3T3‐L1 cells was assessed using Oil Red O (ORO) staining as previously described.^23^ Briefly, on day 7 of differentiation, mature 3T3‐L1 adipocytes were imaged prior to fixing with 4% paraformaldehyde overnight. Fixed cells were then rinsed with deionized (DI) water and subsequently stained with 60% ORO solution diluted with isopropanol for 20 minutes. After removal of the staining solution and rinsing with water, images of stained cells were obtained via an integrated digital camera linked to a Micromaster inverted phase contrast microscope (Thermo Fisher Scientific). The stain uptake by lipid droplets was quantified by eluting ORO stain with 100% isopropanol for 5 minutes, and absorbance was measured at 500 nm using a GloMax‐Multi Detection System (Promega, Madison, WI). These results were normalized with cell lysate total protein concentration using the BCA Protein Assay Kit (ThermoFisher Scientific).

### 2.5 RNA and DNA isolation and semiquantitative RT‐PCR analysis

RNA and DNA isolation and quantification were performed as previously described.^22^, ^23^ Briefly, total RNA and DNA were isolated using TRI reagent (Molecular Research Center) following the manufacturer's instructions. NanoDrop ND‐1000 Spectrophotometer (NanoDrop Technologies) was used to quantify total RNA and DNA abundance. After RNA isolation, reverse transcription was carried out using a High Capacity cDNA Reverse Transcription kit (Thermo Scientific) following the manufacturer's instructions. Target genes and the housekeeping gene, *36b4,* mRNA expression was then measured quantitatively using Power Up SYBR Green Master Mix following the manufacturer's protocol (Applied Biosystems). Similarly, mtDNA abundance was measured via PCR amplification of mtDNA marker COXIIa and subsequently normalized to the mtDNA housekeeping marker, 18S. PCR reactions were run in a 96‐well format using an ABI 7300HT instrument. Relative gene expression was calculated using the 2^(−ΔΔCt)^ method, which was normalized against the housekeeping genes and converted to fold. All primer sequences are listed in the Tables S1‐S3.

### 2.6 Western blot analysis

Western blot analysis was performed as previously described.^22^ Briefly, protein extraction was completed using ice‐cold phosphate buffered saline with phosphatase inhibitor cocktail (ThermoFisher Scientific) to rinse the cells and cell lysate was prepared using 1x radioimmunoprecipitation assay buffer (Cell Signaling, Danvers, MA) with freshly added phenylmethylsulfonyl fluoride (Sigma). Protein concentrations were determined using BCA Protein Assay kit (ThermoFisher Scientific). Total cell lysates were subjected to electrophoresis on 10%‐12% sodium dodecyl sulfate poly‐acrylamide gel and transferred to polyvinylidene difluoride membrane (Bio‐Rad). The membrane was blocked in 20 mmol/L Tris·HCl, 137 mmol/L NaCl, and 0.1% Tween 20 (tris‐buffered saline with tween 20 (TBST) at pH 7.4) containing 5% nonfat milk. The membranes were incubated with diluted primary antibodies at varying times, followed by incubation with the corresponding diluted secondary antibody. Washing steps between antibody incubations included TBST on a rocker four times for 10 minutes. Each membrane was then incubated with SuperSignal West Pico Chemiluminescent Substrate (ThermoFisher Scientific) for 5 minutes. Excess substrate was removed, and the signal was detected using an Odyssey^®^ FC imaging system (Licor Biosciences) or an x‐ray film processor utilizing HyBlot CL autoradiography film (Denville). Membranes were reprobed using stripping buffer (62.5 mmol/L Tris‐HCl, 2% SDS, and 100 mmol/L 2‐mercaptoethanol) at 50°C for 5‐10 minutes. The signal was quantified by densitometry using Odyssey^®^ FC imaging system.

### 2.7 Flux experiment

Seahorse XF24 Analyzer (Agilent Technologies) was used to measure changes in oxygen consumption rate (OCR) in 3T3‐L1 adipocytes. 3T3‐L1 preadipocytes were seeded in a 6‐well format and differentiated in the presence of indicated treatments. On day 6, cells were reseeded (at an identical cell number per well) into XF24 Microplates (Agilent Technologies) in maintenance media only (no treatments were added) for 24 hours. On day 7, cells were treated with ISO (1 μmol/L) without Indo or Rosi treatment for an additional 24 hours. In preparation for cells to undergo mitochondrial stress test, cells were washed three times and incubated with sterile 37°C XF Assay DMEM media with 1 mmol/L sodium pyruvate, 2 mmol/L Glutamax, 10 mmol/L D‐glucose, and 2% bovine serum albumin (pH 7.4 ± 0.1) in 37°C non‐CO_2_ incubator for 1 hour. During this incubation, mitochondrial stress test chemical compound injections were prepared using XF assay buffer in the following manner [final concentration]: Port A, Oligomycin [1.25 μmol/L]; Port B, Carbonyl cyanide‐4 (trifluoromethoxy) phenylhydrazone (FCCP) [5 μmol/L]; and Port C, Rotenone and Antimycin [1 μmol/L]. The diluted compounds were loaded into their respective ports in a prehydrated sensor cartridge. Following sensor cartridge calibration, the calibration plate was replaced with the experimental cell‐plate and the cellular respiration assays were initiated. Respiration calculations were performed as previously described.^25^, ^26^

### 2.8 Transfection and reporter gene assays

Reporter gene assays were performed as previously described.^22^, ^23^, ^27^ Briefly, PPAR transactivation reporters consist of murine PPARγ or PPARα ligand binding domain (LBD) fused with a Gal4 DNA‐binding domain (DBD) (mPPARγ‐Gal4 or mPPARα‐Gal4) and a reporter construct comprised of an upstream activating sequence (UAS) thymidine kinase‐linked luciferase, 4xUAS‐TK‐luc. 3T3‐L1 preadipocytes were seeded one day prior to transfection at 1x10^4^ per well in 48‐well format. The preadipocytes were then transiently transfected with mPPARα‐Gal4 or mPPARγ‐Gal4, 4xUAS‐TK‐Luc (hereon referred to as PPARα or PPARγ‐Gal4‐Luc), and β‐galactosidase (**β‐gal**) control plasmid using Lipofectamine 3000 and PLUS Reagent (ThermoFisher Scientific) for 24 hours. The cells were then subjected to Indo or the controls for an additional 15‐18 hours. Cell lysate was prepared using Reporter Lysis Buffer (Promega) with Luciferase substrate and β‐gal substrate. Luciferase activity and β‐gal were quantified using luminescence or absorbance respectively by a GloMax‐Multi Detection System (Promega).

### 2.9 Lentiviral shRNA particle infection generating stably infected cell pools

PPARγ knockdown (PPARγ‐KD) and scrambled nontargeting control (SCR) in 3T3‐L1 stable cell lines were generated as previously described.^27^ Briefly, 3T3‐L1 preadipocytes were seeded at 50% confluence in 6‐well plates, and subsequently infected with MISSION shRNA lentiviral transduction particles for PPARγ or control (Sigma‐Aldrich) according to the manufacturer's instructions. Puromycin (2 μg/mL) was used to select stably infected cells.

### 2.10 Statistical analysis

Data are presented as mean ± SEM. SigmaPlot 14 (Systat Software, Inc) was used to perform statistical analysis. Detailed statistical analysis is provided in the Supplemental Methods. The level of significance was set at *P* < .05.

## 3 RESULTS

### 3.1 Indomethacin is not cytotoxic to 3T3‐L1 preadipocytes

**FIGURE 1:**
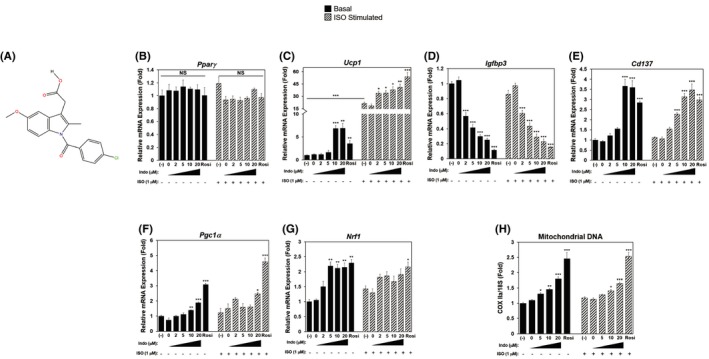
Indomethacin promotes browning as well as mitochondrial biogenesis in 3T3‐L1 adipocytes. Confluent 3T3‐L1 preadipocytes were induced to differentiate in the presence or absence of increasing concentrations of Indo, vehicle control DMSO (Indo 0 µmol/L), or Rosi (1 µmol/L) for 7 d. On day 7, in addition to treatments, cells were stimulated for 6 h with ISO (1 µM) prior to lysis and further analysis. A, Indomethacin chemical structure. B‐G, Relative mRNA gene expression of brown (*Pparγ*, *Ucp1*), beige (*Cd137*), white (*Igfbp3*), and mitochondrial markers (*Pgc1α, Nrf1*), normalized to *36b4*. (H) Mitochondrial DNA, CoxIIa, is normalized to 18S. Each graph is expressed as fold of that of the (‐), non‐ISO‐stimulated sample (set at 1). One‐Way ANOVA was performed followed by multiple comparison tests with Student‐Newman‐Kewls method for treatment and ISO‐responsive relationship. The bar indicates the statistical significance of the non‐ISO group compared to ISO group. Statistical significance is indicated by *, **, ***, *P* < .05, *P* < .01, and *P* < .001, respectively

To eliminate the possibility of potentially harmful effects concerning the multi‐target chemical compound Indo (Figure 1A), 3T3‐L1 preadipocytes were treated with increasing concentrations (0, 2, 5, 10, 20, and 50 μmol/L) of Indo for 24 hours, as described. Utilizing MTT assays, it was determined that there were no significant changes in cell viability, indicating that the range of concentrations of Indo used is not cytotoxic to 3T3‐L1 cells, justifying further experimentation (Figure S1A).

### 3.2 Differentiation of 3T3‐L1 adipocytes is augmented by Indomethacin

Treating 3T3‐L1 cells with Indo throughout the differentiation process led to a significant increase in differentiation, as demonstrated by increased lipid accumulation and induction of differentiation markers. Indo dose‐dependently promoted lipid accumulation as shown by ORO staining, both visually (Figure S1B) and quantitatively (Figure S1C). In addition to lipid accumulation, adipocyte differentiation markers were assessed in mature 3T3‐L1 cells, which also showed significant upregulation in CCAAT enhancer binding protein alpha (*C/ebpα)* and fatty acid binding protein‐4 (*Fabp4)* mRNA expression, while fatty acid synthase (*Fasn)* mRNA expression was down‐regulated (Figure S1D). Protein expression of the differentiation marker perilipin‐1 (PLIN1) and FABP4 was significantly induced (Figure 1E,F).

### 3.3 Induction of mRNA expression of browning markers in 3T3‐L1 adipocytes by Indomethacin

Indo treated 3T3‐L1 adipocytes also had increased expression of brown fat, beige fat, and mitochondrial markers and decreased expression of white adipocyte‐specific markers. Master adipocyte differentiation marker, *Pparγ* mRNA expression was not significantly altered by Indo or Rosi treatment (Figure 1B). mRNA expression of *Ucp1*, the hallmark for browning and brown adipogenesis, was dose‐dependently induced by Indo treatment, which was further augmented by ISO stimulation (Figure 1C). In addition, Indo treatment led to a dose‐dependent suppression of white adipocyte‐specific marker, insulin growth factor binding protein 3 (*Igfbp3)*, both in basal and ISO‐stimulated conditions, with no additional suppression by ISO (Figure 1D). Beige specific marker, tumor necrosis factor receptor superfamily member 9 (*Cd137)*, was also induced by Indo treatment with no additional enhancement by ISO stimulation (Figure 1E).

Furthermore, mitochondrial biogenesis marker, *Pgc1α* mRNA expression was significantly induced by Indo treatment (Figure 1F). mRNA expression of nuclear respiratory factor‐1 (*Nrf1*), a gene encoding for a transcription factor to activate genes required for mitochondrial DNA transcription and replication, was significantly induced by Indo treatment (Figure 1G). Enhanced mitochondrial biogenesis is further confirmed by the relative abundance of mtDNA, which was significantly increased by Indo treatment (Figure 1H).

#### 3.3.1 Indomethacin suppresses PPARγ and ADRB3 protein expression and diminishes response to β‐adrenergic receptor agonist in 3T3‐L1 adipocytes

**FIGURE 2:**
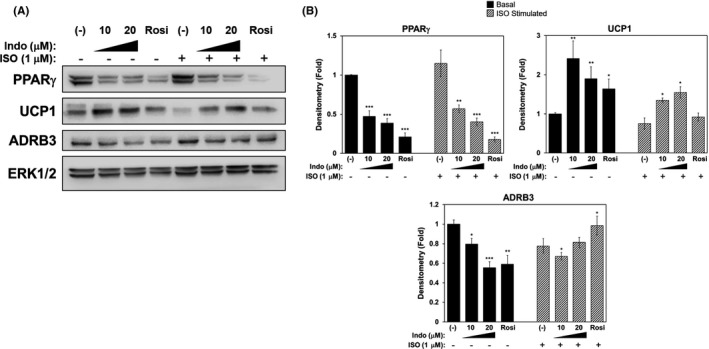
Indomethacin suppresses PPARγ and ADRB3 protein expression in 3T3‐L1 adipocytes. Confluent 3T3‐L1 preadipocytes were induced to differentiate in the presence or absence of increasing concentrations of Indo, vehicle control DMSO (Indo 0 μmol/L), or Rosi (1 μmol/L) for 7 d. On day 7, in addition to treatments, cells were stimulated for 24 h with ISO (1 μmol/L) prior to lysis and further analysis. A, PPARγ, UCP1, and ADRB3 protein expression and their densitometry (B). Densitometry of each protein was normalized to the loading control, total ERK1/2. The (‐), non‐ISO‐stimulated sample was set at fold‐1 for each gene. One‐Way ANOVA was performed followed by multiple comparison tests with Student‐Newman‐Kewls method for treatment and ISO‐responsive relationship. Statistical significance is indicated by *, **, ***, *P* < .05, *P* < .01, and *P* < .001, respectively

**FIGURE 3:**
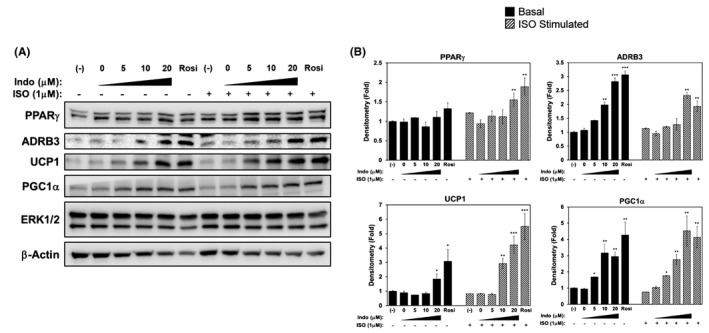
Timely removal of indomethacin and rosiglitazone rescues the response to β‐adrenergic receptor agonist (ISO) in 3T3‐L1 adipocytes. Confluent 3T3‐L1 preadipocytes were induced to differentiate in the presence or absence of increasing concentrations of Indo, vehicle control DMSO (Indo 0 μmol/L), or Rosi (1 μmol/L) for 6 d. On day 6, the cells were replenished with cell media (without Indo or Rosi treatments) for 24 h. On day 7, the cells were stimulated with ISO (1 μmol/L) or vehicle control for an additional 24 h. A, Protein expression of rescued adipocyte genes (ADRB3 and PPARγ), browning marker (UCP1), and mitochondrial biogenesis marker (PGC1α) and their densitometry (B). Densitometry of each protein was normalized to loading control, ERK1/2 with the (‐), non‐ISO‐stimulated sample, set at fold‐1 for each gene. One‐Way ANOVA was performed followed by multiple comparison tests with Student‐Newman‐Kewls method for treatment and ISO‐responsive relationship. The bar indicates the statistical significance of the non‐ISO group compared to ISO group. Statistical significance is indicated by *, **, ***, *P* < .05, *P* < .01, and *P* < .001, respectively

Indo treated 3T3‐L1 adipocytes also significantly induced UCP1 protein expression under basal and ISO‐stimulated conditions; however, the dose‐dependent and ISO‐stimulated response seen in mRNA expression (Figure 1C) was not reflected in the protein expression (Figure 2A,B). Additionally, treatment of 3T3‐L1 adipocytes with Indo and Rosi throughout the entire differentiation process revealed a significant dose‐dependent suppression of PPARγ protein expression (Figure 2A,B). Due to the muted response to ISO stimulation in the Indo‐ and Rosi‐treated‐3T3‐L1 adipocytes as seen in the protein expression and lack of increase in oxygen consumption measured by Seahorse XF Analyzer (data not shown), protein expression of the β‐3 adrenergic receptor (ADRB3) was determined (Figure 2A,B). ADRB3 is a primary adrenoceptor mediating norepinephrine signaling for thermogenesis in brown adipocytes,^4^ thus being the critical mediator for the ISO‐stimulated response. In this case, the ADRB3 protein expression was decreased by Indo and Rosi treatment as compared to the vehicle control in the basal and ISO‐stimulated conditions (Figure 2A,B). Interestingly, removal of Indo and Rosi 24 hours prior to ISO stimulation successfully recovered the protein expression of both PPARγ and ADRB3 in both basal and ISO‐stimulated conditions (Figure 3A,B).

Moreover, removal of Indo and Rosi 24 hours prior to the ISO stimulation also led to increased protein expression of brown markers, which were more consistent with their corresponding mRNA expression in both basal and ISO‐stimulated conditions. As shown in Figure 3, Indo significantly induced UCP1 and PGC1α protein expression in both basal and ISO‐stimulated conditions (Figure 3A,B). In addition, protein expression of other mitochondrial structural markers were assessed, including VDAC1, CYTC, and MNSOD, which were all dose‐dependently increased in response to Indo in both basal and ISO‐stimulated conditions (Figure S2A,B).

### 3.4 Enhanced flux capacity of 3T3‐L1 adipocytes by indomethacin

**FIGURE 4:**
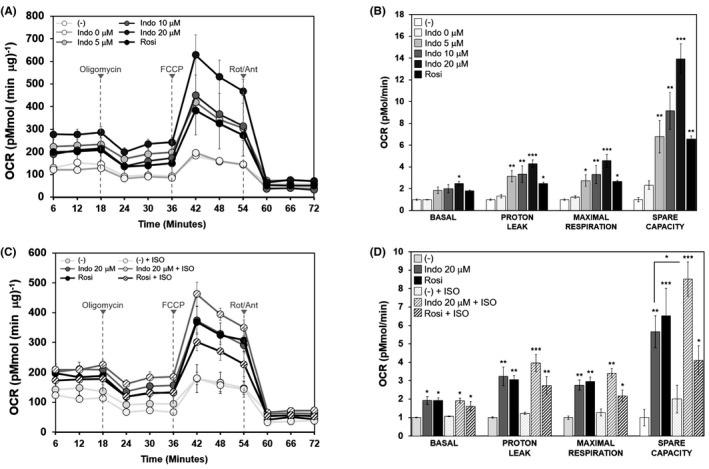
Indomethacin enhances mitochondrial respiration and uncoupling in 3T3‐L1 adipocytes. Confluent 3T3‐L1 preadipocytes were induced to differentiate in the presence or absence of increasing concentrations of Indo, vehicle control DMSO (Indo 0 μmol/L), or Rosi (1 μmol/L) for 6 d. On day 6, cells were evenly reseeded into an XF 24 well plate. A and B, Reseeded cells were maintained in the presence of Indo and Rosi treatment until initiation of flux measurements on day 8. C and D, To restore the response to ISO, reseeded cells were maintained in the absence of Indo or Rosi for 24 h. Cells were then treated with ISO (1 μmol/L) or vehicle for 24 h prior to initiation of flux measurements. Respiration calculations (B and D) were performed, and are displayed so that the (‐), non‐ISO sample average is set at fold‐1 for each type of respiration calculation group. Statistical differences in C and D are treatment compared to its (‐) control (either (‐) or (‐)+ISO). The bar indicates the statistical significance of the non‐ISO group compared to ISO group. ANOVA with repeated measures and one‐way ANOVA was performed to show statistical differences between treatment and controls indicated by *, **, ***, *P* < .05, *P* < .01, and *P* < .001, respectively

To assess the competency of these brown‐like adipocytes, differentiated 3T3‐L1 adipocytes treated with or without increasing concentrations of Indo underwent flux measurements using the Seahorse XF Analyzer. Mitochondrial stress tests revealed that Indo treated 3T3‐L1 adipocytes not only had greater basal OCR, but also had increased proton leak, maximal respiration, and spare respiratory capacity (Figure 4A,B). In order to restore ADRB3 protein expression and the subsequent flux response to ISO, Indo and Rosi were removed 24 hours prior to ISO stimulation. ISO stimulation led to significantly enhanced spare respiratory capacity, but did not significantly change proton leak or maximal respiration increased by Indo or Rosi (Figure 4C,D).

### 3.5 Indomethacin activates PPARγ in 3T3‐L1 cells

**FIGURE 5:**
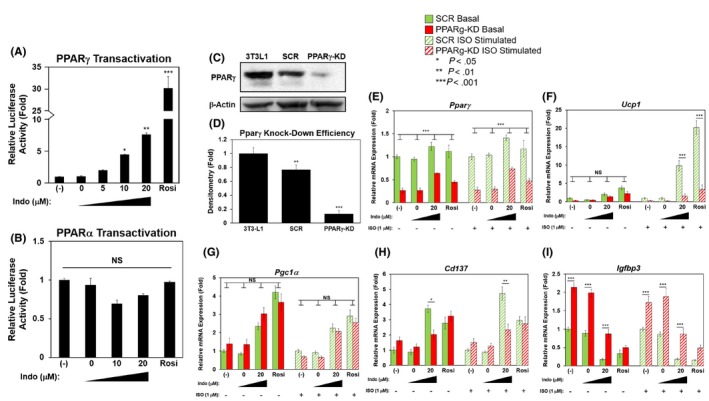
Indomethacin dose‐dependently activates PPARγ, knockdown of which attenuates browning. A and B, 3T3‐L1 preadipocytes were transiently transfected with PPARγ‐Gal4 (A) or PPARα‐Gal4 (B), Gal4‐Luc and β‐gal for 24 h, and then treated with increasing concentrations of Indo for an additional 18 h. Luciferase activity was normalized to β‐gal activity, the nontreated (‐) sample was set at fold‐1. C, 3T3‐L1 preadipocytes were stably infected with lentiviral shRNA particles targeting PPARγ or a scrambled control (SCR). Knockdown (KD) efficiency was determined by western blot analysis. D, Densitometry of PPARγ protein expression was normalized to the loading control, β‐Actin, with the parental 3T3‐L1 cells set as fold‐1. E‐I, Confluent 3T3‐L1 cells with stable knockdown of PPARγ (PPARγ‐KD) or nontargeting control (SCR) were induced to differentiate in the presence of absence of Indo, vehicle control DMSO (Indo 0 μmol/L), or Rosi (1 μmol/L) for 7 d. Relative mRNA expression of *Pparγ*, *Ucp1, Pgc1α*, *Cd137*, and *Igfbp3* normalized to *36b4* are shown, the (‐)‐non‐ISO‐stimulated SCR sample and (‐)‐ISO‐stimulated SCR sample were both set at fold‐1 for each group, independently, for easy comparison. One‐Way ANOVA was performed followed by multiple comparison tests with Student‐Newman‐Kewls method to determine significant differences between the SCR and PPARγ‐KD of the same treatment. The bar indicates the statistical significance between PPARγ‐KD and SCR for each treatment. Statistical significance is indicated by *, **, ***, *P* < .05, *P* < .01, and *P* < .001, respectively

Indo has previously been reported to activate PPARγ in CV‐1^14^ and HeLa cells.^15^ Consistently, Indo significantly activated PPARγ in a dose‐dependent manner in 3T3‐L1 preadipocytes using PPARγ transactivation assays (Figure 5A). This activity was not mirrored when testing PPARα transactivation, further demonstrating its specificity for PPARγ by Indo at the tested doses (Figure 5B).

### 3.6 Browning induced by indomethacin is attenuated by PPARγ knockdown

To confirm Indo's ability to operate as a browning agent via its action as a PPARγ‐agonist, PPARγ knockdown (PPARγ‐KD) was generated in 3T3‐L1 cells. PPARγ protein expression analysis confirmed 86% knockdown in the selected KD cell pools compared to the parental cells (Figure 5C,D). PPARγ‐KD cells and nontargeting control (SCR) cells were differentiated in the presence of Indo (20 μmol/L), Rosi (1 μmol/L), or vehicle control, DMSO (Indo 0 μmol/L). Consistent with the protein expression, there was significant reduction of *Pparγ* mRNA expression in the KD cells as compared to the SCR cells in both basal and ISO‐stimulated conditions, with no significant differences between the treatments (Figure 5E). Upregulation of *Ucp1* mRNA expression by Indo was significantly suppressed in the PPARγ‐KD as compared to the SCR cells (Figure 5F). There were no significant differences in *Pgc1α* mRNA expression concerning Indo or vehicle control treated PPARγ‐KD cells as compared to the SCR cells (Figure 5G). The upregulation of mRNA expression of beige fat‐specific marker, *Cd137*, by Indo was significantly attenuated in PPARγ‐KD cells compared to the SCR cells (Figure 5H). Knockdown of PPARγ upregulated the basal mRNA expression of the white fat‐specific marker, *Igfbp3* (Figure 5I). Suppression of *Igfbp3* mRNA expression by Indo was significantly hindered in PPARγ‐KD cells compared to the SCR cells in both basal and ISO‐stimulated conditions (Figure 5I).

### 3.7 Indomethacin induces browning in primary stromal cells derived from human subcutaneous fat

**FIGURE 6:**
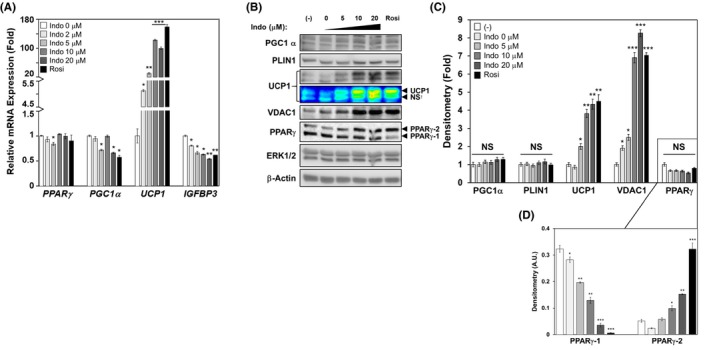
Indomethacin induces browning in primary stromal cells derived from human subcutaneous fat. Human primary stromal cells from subcutaneous fat were induced to differentiate in the presence or absence of increasing concentrations of Indo, vehicle control DMSO (Indo 0 μmol/L), or Rosi (1 μmol/L) for 14 d. A, mRNA gene expression of *PPARγ*, *PGC1α, UCP1,* and *IGFBP3*, normalized to human *36B4*. B, PGC1α, PLIN1, UCP1, VDAC, and total PPARγ protein expression and their densitometry (C). D, Densitometry of PPARγ‐1 and PPARγ‐2 was quantified separately and graphed. All densitometric comparison of the average protein expression is normalized to that of loading control, total ERK1/2. Unless otherwise noted, each graph is expressed as fold of that of the Indo 0 μmol/L (mRNA) or (‐) (protein) sample for each gene (set as fold‐1). Densitometry shown in (D) is set using A. U. (Arbitrary Units). One‐Way ANOVA was performed followed by multiple comparison tests with Student‐Newman‐Kewls method for concentration‐responsive relationships. The bar indicates the significant or nonsignificant (NS) state for individual samples compared to their control within each gene group. NS^†^: Nonspecific band. Statistical significance is indicated by *, **, ***, *P* < .05, *P* < .01, and *P* < .001, respectively

The browning effects demonstrated in 3T3‐L1 adipocytes is similarly apparent in HPsubQ adipocytes treated with Indo. Indo dose‐dependently increased brown fat marker, *UCP1* mRNA expression (Figure 6A), with simultaneous suppression of white fat‐specific marker, *IGFBP3* mRNA expression (Figure 6A). Similar to what was previously shown in 3T3‐L1 cells, there is no significant change in *PPARγ* mRNA expression (Figure 6a). In contrast, there was no significant increase, but rather a decrease, in *PGC1α* mRNA expression (Figure 6A).

Similarly, PGC1α and PLIN1 protein expression did not show a significant response to Indo treatment (Figure 6B,C). However, Indo dose‐dependently increased UCP1 and VDAC1 protein expression in response to increasing concentrations of Indo (Figure 6B,C). There were no significant differences in total PPARγ protein expression among the treatments (Figure 6B,C); however, analysis of individual PPARγ isoforms revealed a significant dose‐dependent suppression of PPARγ‐1 and a reciprocal dose‐dependent enhancement in PPARγ‐2 protein expression by increasing concentrations of Indo (Figure 6B,D).

### 3.8 Indomethacin promotes brown adipogenesis in human brown cell line

**FIGURE 7:**
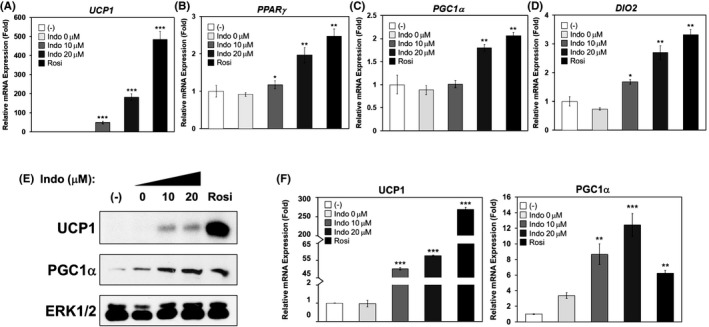
Indomethacin promotes brown adipogenesis in human brown fat cell line. Human brown fat cells were induced to differentiate in the presence or absence of increasing concentrations of Indo and vehicle control DMSO (Indo 0 μmol/L) for 21 d. A‐D, Relative mRNA gene expression of *PPARγ*, *PGC1α, UCP1,* and *DIO2*, all normalized to human *36B4*. E, UCP1 and PGC1α protein expression and their densitometry (F). Densitometry of UCP1 and PGC1α protein was normalized to the loading control, total ERK1/2. Each graph is expressed as fold of that of the Indo 0 μmol/L sample (set as fold‐1). One‐Way ANOVA was performed followed by multiple comparison tests with Student‐Newman‐Kewls method for concentration‐responsive relationship. Statistical significance is indicated by *, **, ***, *P* < .05, *P* < .01, and *P* < .001, respectively

A previous study demonstrated that Indo promotes brown adipogenesis in murine brown cell line and C57Bl/6J mice when Indo is delivered via an osmotic pump directly onto the BAT.^22^ In this study, a HBr cell line was used to further investigate the potential for Indo to promote brown adipogenesis in human brown fat cells. The results demonstrate that Indo dose‐dependently induced UCP1 mRNA and protein expression in HBr adipocytes (Figure 7A,E,F). Moreover, Indo dose‐dependently increased mRNA expression of BAT‐enriched markers, *PPARγ*, *PGC1α*, and deiodinase iodothyronine type II (*DIO2)* (Figure 7A‐E). PGC1α protein expression was also dose‐dependently increased by Indo (Figure 7E,F).

## 4 DISCUSSION

For the first time, we report herein the dose‐dependent browning effects of Indo on murine and human white adipocytes and human brown adipocytes.

Our findings that Indo activates PPARγ and promotes 3T3‐L1 adipocyte differentiation are consistent with previous reports demonstrating that TZDs and Indo promote white adipocyte differentiation in vitro and in vivo.^15^, ^21^, ^28^, ^29^ Moreover, we show that Indo also significantly induced browning in 3T3‐L1 adipocytes as shown by enhanced UCP1 mRNA and protein expression as well as beige fat‐specific marker, *Cd137*, with simultaneous suppression of white fat‐specific marker, *Igfbp3*. It is worth noting that continuous treatment of Indo increased *Ucp1* mRNA expression under basal and ISO‐stimulated conditions; however, removal of Rosi and Indo for 24 hours prior to ISO stimulation is required to restore the cellular response to ISO as it relates to UCP1 protein expression. This is likely due to the fact that continuous PPARγ activation by Rosi or Indo suppressed protein expression of PPARγ and ADRB3, which are the upstream activator of UCP1 and the receptor for ISO, respectively.

The downregulation of PPARγ protein expression by Rosi and Indo may be explained by self‐regulation, feedback inhibition, and/or protection of the receptors from overutilization.^30^ The fact that adipogenesis was not inhibited, but rather enhanced by continuous treatment with Rosi or Indo, may suggest that there is a threshold of receptor expression required for sufficient differentiation of adipocytes.^31^ Furthermore, Indo dose‐dependently suppressed ADRB3 protein expression in 3T3‐L1 adipocytes. These results are consistent with a previous report which demonstrated that treatment of troglitazone, a TZD, led to acute suppression of *Adrb3* mRNA levels in HIB‐1B murine brown fat cells, which was accompanied by decreased response to various β‐AR agonists.^32^ Adjustment of the differentiation program by removal of Rosi or Indo for 24 hours prior to ISO stimulation led to recovery and subsequent increases of both PPARγ and ADRB3 protein expression (Figure 3A,B). Consequently, there was an enhancement in UCP1 protein expression in response to ISO stimulation, which was accompanied by similar protein expression of other mitochondrial markers (PGC1α, VDAC1, CYTC, MNSOD) (Figure 3A,B, Figure S2A,B).

Among the critical differences between brown or brown‐like adipocytes and white adipocytes, is the relative abundance of functional mitochondria expressing UCP1.^7^, ^8^, ^9^, ^10^ Our results demonstrate Indo's ability to enhance synthesis, quantity, and function of adipocyte mitochondria, as shown by increased mRNA and/or protein expression of mitochondrial marker genes, mtDNA, and mitochondrial respiration. Regulation of quality and quantity of mitochondria is a tightly controlled and highly coordinated process.^33^, ^34^, ^35^ Master regulator of mitochondrial biogenesis, PGC1α, interacts with the mitochondrial‐gene transcription factor, NRF1, to further promote mitochondrial biogenesis and leads to increased expression of mitochondrial enzymes and structural components, such as COX4A.^35^ mRNA expression of BAT‐enriched mitochondrial markers *Pgc1α* and *Nrf1* was dose‐dependently increased in response to Indo treatment in 3T3‐L1 cells. Together with the elevated mtDNA abundance, these results suggest an overall increase of mitochondrial quantity, which is accompanied by higher UCP1 expression.

To further confirm the effects of Indo on mitochondrial respiration, mitochondrial stress tests were performed. It was found that Indo dose‐dependently increased basal OCR, proton leak (ie, respiration uncoupling, an indication of UCP1 function, which is consistent with increased UCP1 protein expression by Indo), and maximal and spare respiratory capacity in 3T3‐L1 adipocytes under basal (non‐ISO‐stimulated) conditions. Under ISO‐stimulated conditions, only spare respiration was significantly augmented by Indo. Increased basal OCR and proton leak could merely indicate physiological adaptation or a response to stress^34^; however, these indices in combination with increased maximal and spare respiratory capacity indicate that Indo‐treated adipocytes have a larger and healthier network of mitochondria with a heightened ability to withstand oxidative damage and stress.^34^ Therefore, together with gene expression data, our results demonstrate that Indo promotes browning with a larger population of healthy and functional mitochondria.

Although primarily recognized as an NSAID, which inhibits COX‐1 and COX‐2, Indo has also been shown to act as a PPARγ‐agonist in several cell types, including CV‐1^14^ and HeLa cells.^15^ Consistently, our results show that Indo dose‐dependently activates PPARγ, but not PPARα, in 3T3‐L1 preadipocytes as shown by respective PPAR transactivation assays. Several studies have suggested that Indo's effects may vary based on concentration. It was shown that Indo acts as a PPARγ agonist at higher concentrations (10‐100 μmol/L)^14^, ^15^ whereas it only has COX‐inhibiting effects at lower concentrations (0.1‐1.5 μmol/L).^36^ Studies have demonstrated that COX‐2 expression/activity is required for UCP1 expression and browning of white adipocytes.^37^ Therefore, it is conceivable that Indo's browning effects seen here are not due to its COX inhibition capacity.

As previously mentioned, many reports demonstrate Rosi as a robust adipocyte “browning agent” via its action as a potent full PPARγ agonist,^38^, ^39^ at least in part by induction of PGC1α expression.^3^ Therefore, it is possible that Indo's browning effects may work through the PPARγ‐PGC1α axis as well. To investigate the role of PPARγ in Indo's browning effects, PPARγ knockdown (KD) in 3T3‐L1 cells was generated and differentiated with or without Indo. These results revealed that the upregulated mRNA expression of *Ucp1* and *Cd137* by Indo was significantly attenuated in PPARγ‐KD cells as compared to the control (SCR) cells. In contrast, there was a significantly hindered downregulation of *Igfbp3* mRNA expression by Indo in PPARγ‐KD cells. However, there were minimal differences in *Pgc1α* mRNA expression between PPARγ‐KD and SCR cells, suggesting that the effects of Indo on *Pgc1α* mRNA expression are independent of PPARγ activation by Indo. These results suggest that Indo's capacity to induce browning in 3T3‐L1 cells is due to its PPARγ‐agonist activity and is independent of PGC1α.

Indo's browning effects extend into human primary stromal cells derived from the subcutaneous adipose tissue. This Indo‐induced browning is demonstrated by the dose‐dependent increase in UCP1 mRNA and protein expression as well as dose‐dependent suppression of white fat‐specific marker, *IGFBP3*, mRNA expression in HPsubQ adipocytes. Moreover, Indo also led to increased protein expression of VDAC1, a major mitochondrial structural component. However, there was no significant upregulation in PGC1α mRNA or protein expression in HPsubQ adipocytes as compared to 3T3‐L1 adipocytes, suggesting that Indo's browning effects in HPsubQ adipocytes may also be PGC1α‐independent.

Moreover, HPsubQ cells showed differential expression of the PPARγ isoforms in response to Indo. When both PPARγ‐1 (53 kDa) and PPARγ‐2 (57 kDa) were analyzed together, there were no differences by Indo treatments; however, when assessed separately, it became evident that Indo led to a significant dose‐dependent increase in PPARγ‐2 and reciprocal dose‐dependent decrease of PPARγ‐1 protein expression in HPsubQ adipocytes. Although the literature on the differential expression of PPARγ isoforms is dearth and controversial, a study of 50 obese female patients demonstrated that PPARγ‐2, an adipose‐specific isoform, has overall higher expression compared to PPARγ‐1, in both visceral adipose tissue and subcutaneous adipose tissue.^40^ Moreover, it was found that PPARγ‐2 is inversely correlated with BMI and weight, and that higher expression of PPARγ‐1 in SAT is associated with higher circulating insulin levels.^40^ Therefore, an inverse relationship of PPARγ‐1 and PPARγ‐2 elicited by Indo suggests that Indo induces beneficial profiles of PPARγ isoforms in HPsubQ adipocytes, which may be associated with enhanced insulin sensitivity.^40^ Further studies are needed to confirm Indo's effects on insulin sensitivity in HPsubQ adipocytes.

Lastly, Indo significantly enhanced brown adipogenesis in the human brown fat cell line, as demonstrated by increased UCP1 and PGC1α mRNA and protein expression as well as increased *PPARγ* and *DIO2* mRNA expression. Future studies are needed to examine Indo's effects on mitochondrial respiration and uncoupling in the human brown adipocytes.

Known as insulin sensitizers, Rosi and some other TZDs have been used to treat T2DM. However, their clinical uses are limited due to adverse side effects, such as weight gain, bone loss, and congestive heart failure.^41^ Greater understanding of the PPARγ signaling pathways has offered hope to generate improved therapeutics with less side effects.^41^ As an NSAID, Indo's clinical use is limited to inflammatory conditions, adverse side effects have been reported in various systems and organs, including gastrointestinal toxicity.^42^ Therefore, it remains to be determined whether Indo or Rosi can be clinically used as browning agents for human obesity treatment and prevention.

In conclusion, for the first time, the results presented here suggest that Indo, possibly through its PPARγ‐agonist activity, promotes browning in murine and human white adipocytes as well as enhances brown adipogenesis in human brown adipocytes. Increased UCP1 protein expression seen in 3T3‐L1 adipocytes is accompanied by increased mitochondrial biogenesis and uncoupling, as well as enhanced maximal and spare respiratory capacity. In light of Indo's potential to induce browning and brown adipogenesis, and taking into consideration the possible contraindications and adverse side effects, it may be beneficial to explore the possibilities of local delivery of Indo as a therapeutic agent for obesity treatment and prevention.

## DISCLOSURE

The authors have no conflict of interest to declare.

## AUTHOR CONTRIBUTIONS

Participated in research design: Overby, Wang, and Zhao. Conducted experiments: Overby, Yang, and Xu. Performed data analysis: Overby, Yang, and Xu. Wrote or contributed to the writing of the manuscript: Overby, Wang, and Zhao.

## Supporting information

Fig S1‐S2

Table S1‐S3
