# Supplementary figures and images for: Indomethacin promotes browning and brown adipogenesis in both murine and human fat cells
Source: Pharmacol Res Perspect. 2020 May 19;8(3):e00592. doi: 10.1002/prp2.592 (PMC7237299; doi:10.1002/prp2.592)

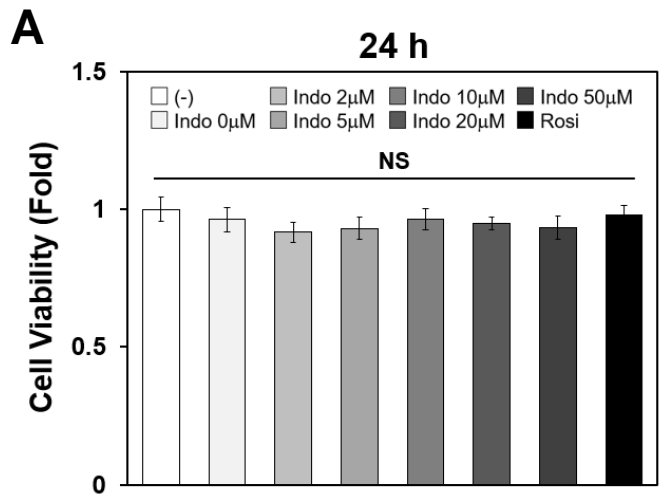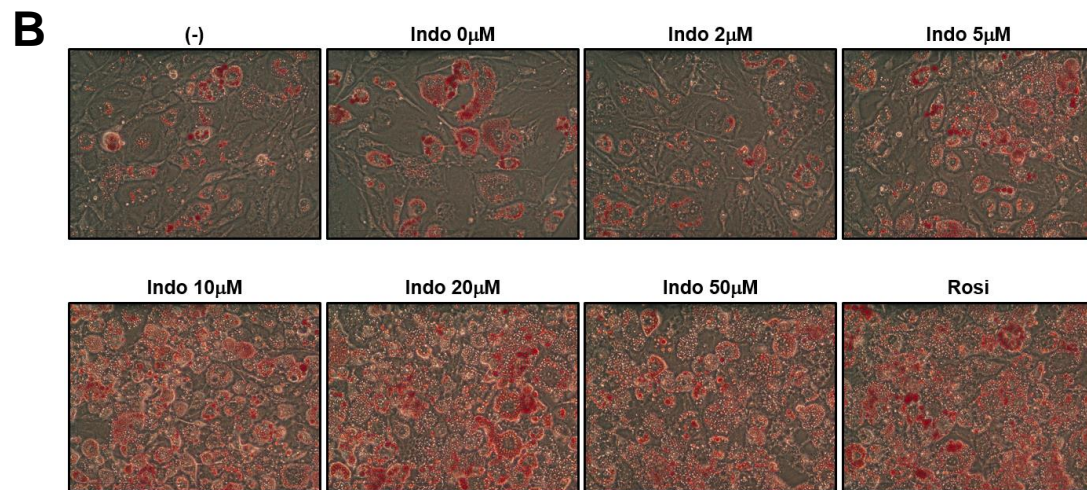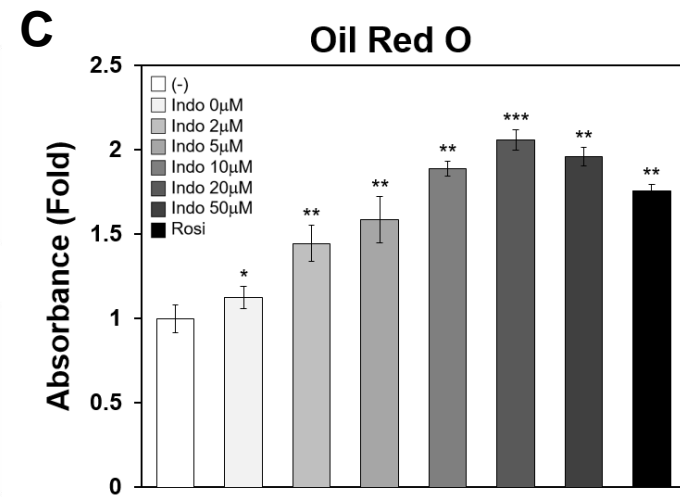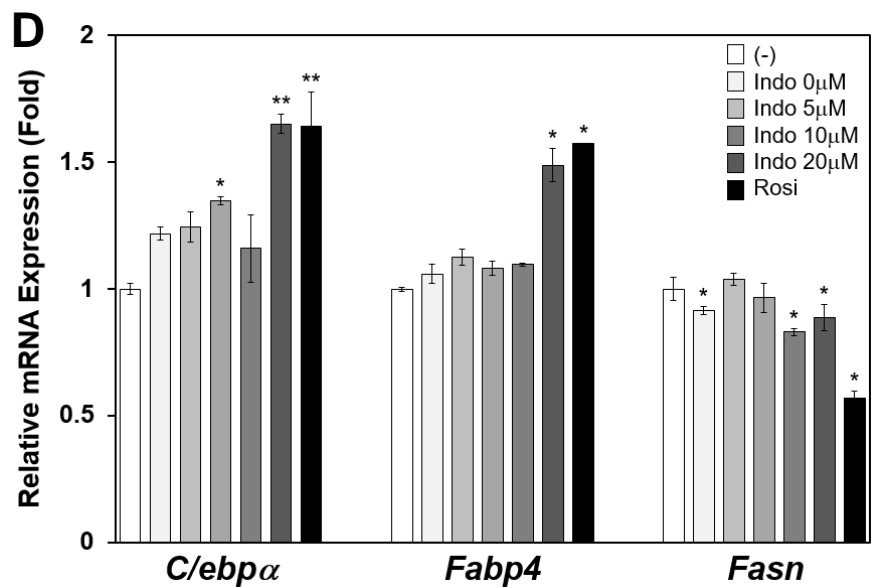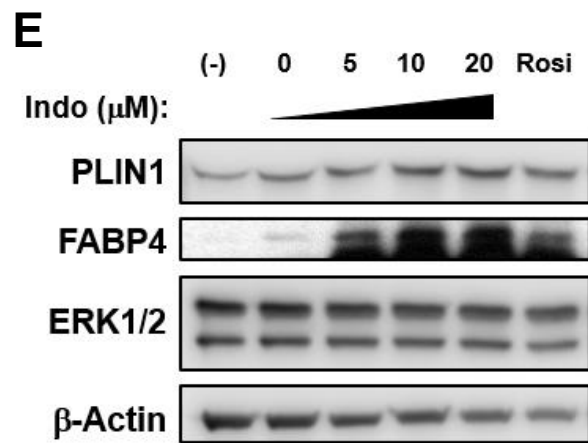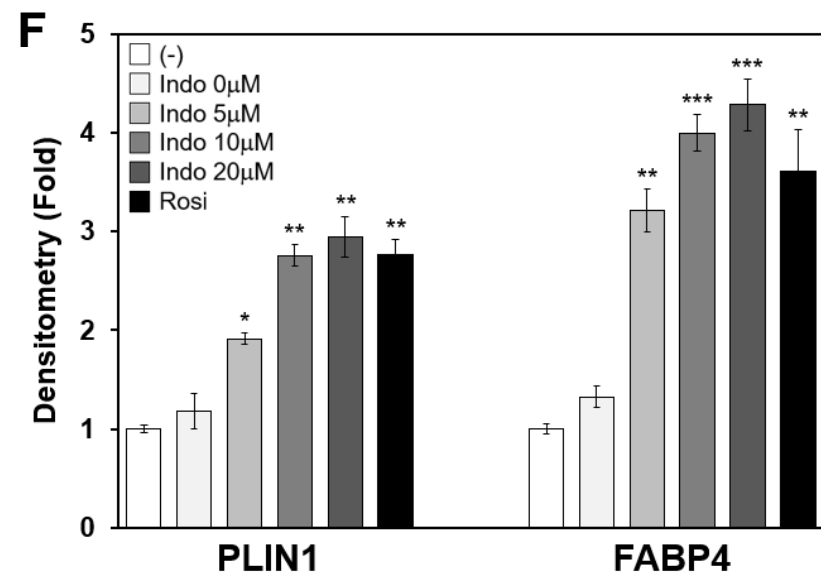

**A**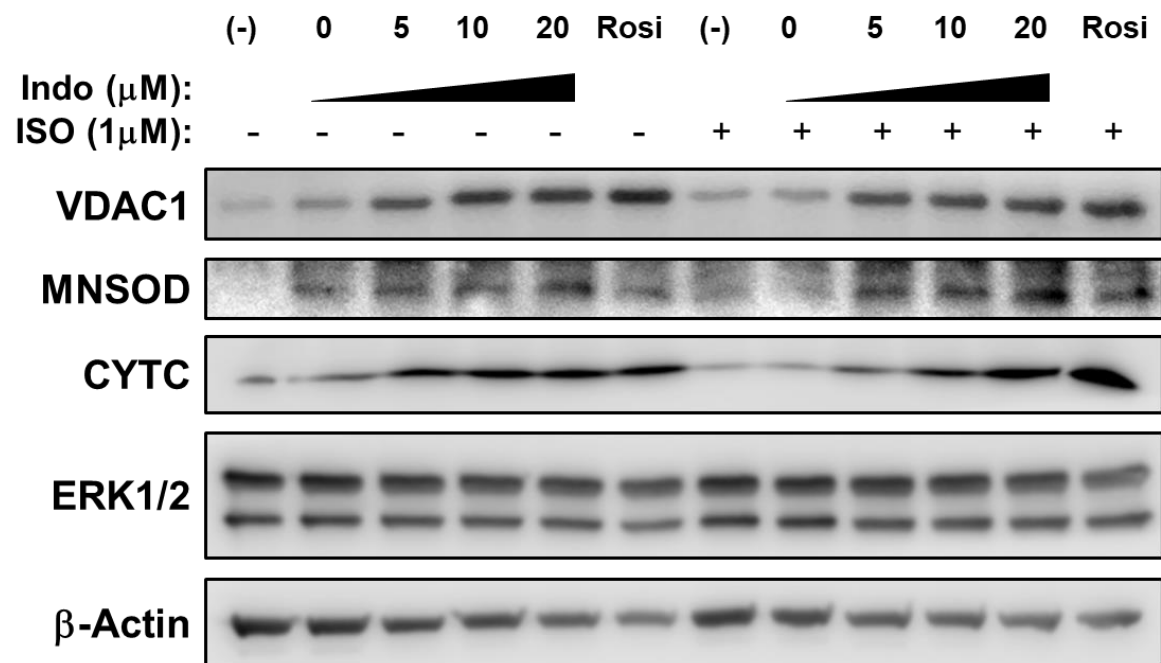**B**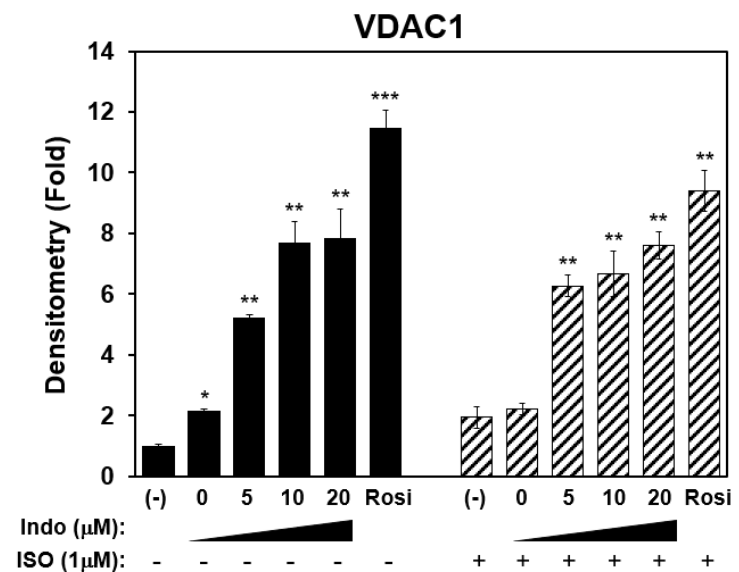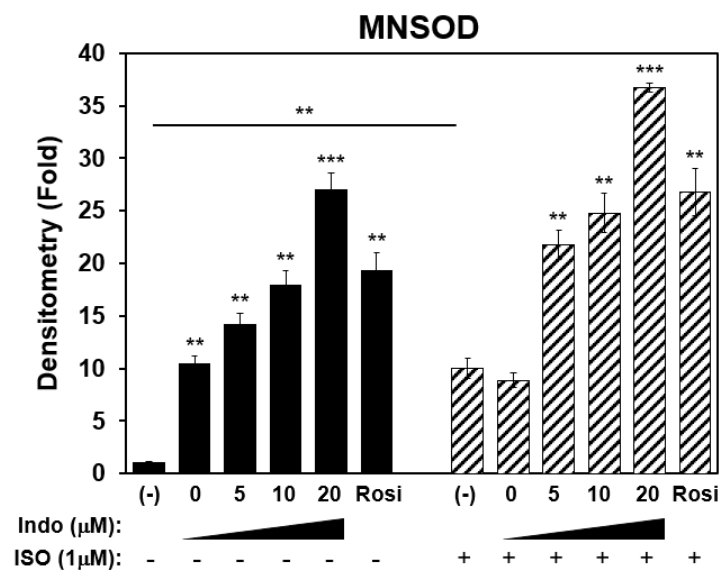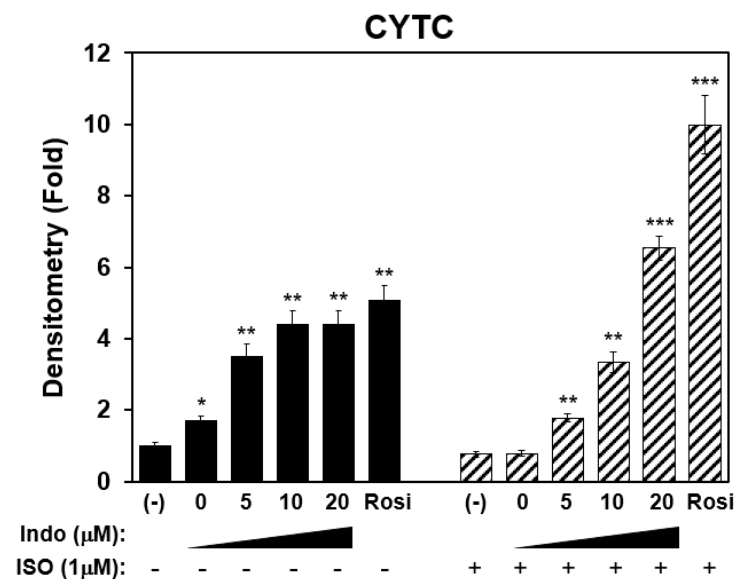

Supplement: Supplementary file 1 — Fig S1‐S2 [file PRP2-8-e00592-s001.pdf]
