# Supplementary material for: Indomethacin promotes browning and brown adipogenesis in both murine and human fat cells
Source: Pharmacol Res Perspect. 2020 May 19;8(3):e00592. doi: 10.1002/prp2.592 (PMC7237299; doi:10.1002/prp2.592)
Supplement: Supplementary file 2 — Table S1‐S3 [file PRP2-8-e00592-s002.docx]

**Table 1. Murine Primer Sequences for qRT-PCR using SYBR green.**

| **Gene name** | **Primer sequences** | |
| --- | --- | --- |
| *36b4* | Forward | GCTTCGTGTTCACCAAGGAGGA |
|  | Reverse | GTCCTAGACCAGTGTTCTGAGC |
| *Fabp4* | Forward | TGAAATCACCGCAGACGACAGG |
|  | Reverse | GCTTGTCACCATCTCGTTTTCTC |
| *β-AR3* | Forward | AGGCACAGGAATGCCACTCCAA |
|  | Reverse | GCTTAGCCACAACGAACACTCG |
| *Cd137* | Forward | CGTGCAGAACTCCTGTGATAAC |
|  | Reverse | GTCCACCTATGCTGGAGAAGG |
| *C/ebpα* | Forward | GCAAAGCCAAGAAGTCGGTGGA |
|  | Reverse | CCTTCTGTTGCGTCTCCACGTT |
| *Cidea* | Forward | GGTGGACACAGAGGAGTTCTTTC |
|  | Reverse | CGAAGGTGACTCTGGCTATTCC |
| *Cox4a* | Forward | TCATTGGCTTCACTGCGCTCGT |
|  | Reverse | TCCAGCATTCGCTTGGTCTGCA |
| *Fasn* | Forward | CACAGTGCTCAAAGGACATGCC |
|  | Reverse | CACCAGGTGTAGTGCCTTCCTC |
| *Igfbp3* | Forward | GTCGCAAATAATGGTGCGCT |
|  | Reverse | GAAAAACTGCCTTCCCGCAG |
| *Nrf1* | Forward | GGCAACAGTAGCCACATTGGCT |
|  | Reverse | GTCTGGATGGTCATTTCACCGC |
| *Pgc1α* | Forward | GAATCAAGCCACTACAGACACCG |
|  | Reverse | CATCCCTCTTGAGCCTTTCGTG |
| *Pparγ* | Forward | GTACTGTCGGTTTCAGAAGTGCC |
|  | Reverse | ATCTCCGCCAACAGCTTCTCCT |
| *Ucp1* | Forward | GCTTTGCCTCACTCAGGATTGG |
|  | Reverse | CCAATGAACACTGCCACACCTC |

| **Gene name** | **Primer sequences** | |
| --- | --- | --- |
| *36B4* | Forward | GCTTCGTGTTCACCAAGGAGGA |
|  | Reverse | GTCCTAGACCAGTGTTCTGAGC |
| *β-AR3* | Forward | AGCCCAGGCTTTGCCAACGGC |
|  | Reverse | GGGACTCATTCAGAACAGAGGC |
| *DIO2* | Forward | AGTGCAGAAGGAGGTGACAACAGT |
|  | Reverse | AAAGTCAAGAAGGTGGCATGTGGC |
| *IGFBP3* | Forward | CGCTACAAAGTTGACTACGAGTC |
|  | Reverse | GTCTTCCATTTCTCTACGGCAGG |
| *PGC1α* | Forward | AGTGGTGCAGTGACCAATCA |
|  | Reverse | CTGCTAGCAAGTTTGCCTCA |
| *PPARγ* | Forward | AGCCTGCGAAAGCCTTTTGGTG |
|  | Reverse | GGCTTCACATTCAGCAAACCTGG |
| *PRDM16* | Forward | CAGCCAATCTCACCAGACACCT |
|  | Reverse | GTGGCACTTGAAAGGCTTCTCC |
| *UCP1* | Forward | AGTTCCTCACCGCAGGGAAAGA |
|  | Reverse | GTAGCGAGGTTTGATTCCGTGG |

**Table 2. Human Primer Sequences for qRT-PCR using SYBR green.**

**Table 3. Murine Mitochondrial DNA Primer Sequences for qRT-PCR using SYBR green.**

| **Gene name** | **Primer sequences** | |
| --- | --- | --- |
| Mouse mt*Cox-II* | Forward | GCTCTCCCCTCTCTACGCAT |
|  | Reverse | AGCAGTCGTAGTTCACCAGG |
